# Heat shock affects the Ca^2+^/calmodulin-dependent protein kinase II dynamic during bovine sperm capacitation and acrosome reaction

**DOI:** 10.3389/fcell.2025.1552282

**Published:** 2025-04-02

**Authors:** Thais de Sousa Santos, Isabelle Scarpini Cotrim, Daniela Franco da Silva, Mayra Elena Ortiz D’Avila Assumpção, Fabiola Freitas de Paula-Lopes, Weber Beringui Feitosa

**Affiliations:** 1 Department of Biological Sciences, Federal University of Sao Paulo, Diadema, Sao Paulo, Brazil; 2 Institute of Biosciences, State University of Sao Paulo, Botucatu, Sao Paulo, Brazil; 3 Department of Animal Reproduction, University of Sao Paulo, Sao Paulo, Sao Paulo, Brazil

**Keywords:** acrosomal membrane, heat stress, spermatozoa, phosphorylation, environment

## Abstract

**Background:**

Heat shock during sperm capacitation affects the spermatozoa quality, resulting in increased early acrosome reaction and consequently decreasing their fertilizing capacity. Although the mechanisms involved in the regulation of sperm capacitation and acrosome reaction are not fully understood, it has been reported that Ca^2+^/calmodulin-dependent protein kinase II (CaMKII) is an important regulator of these processes. Thus, the present aimed to evaluate the effect of heat shock in the CaMKII signaling during the bovine sperm capacitation and acrosome.

**Methods:**

Bovine spermatozoa were *in vitro* capacitated for 4 hours. The acrosome reaction was induced by exposure to heparin and calcium ionophore A23187 for 1 hour. Heat shock was applied by incubating spermatozoa at 41 °C with 7% CO_2_, while the control group was maintained at 38.5 °C with 5% CO_2_. At the end of each treatment, the localization of total CaMKII and phosphorylated CaMKII (pCaMKII), as well as acrosomal membrane integrity, were evaluated by immunofluorescence.

**Results:**

It was observed that CaMKII and not phosphorylated CaMKII (*pCaMKII*) localization at the acrosome region was affected by sperm capacitation. In contrast, the localization of both, CaMKII and its phosphorylated form was affected by the acrosome reaction (p < 0.05). The acrosome membrane integrity, as well as the *p*CamKII localization in bovine spermatozoa, was affected by incubation time. This effect of incubation time was stronger in heated shock sperm, although it was observed only after 2 h of incubation. Heat shock also affected the acrosomal localization of *p*CaMKII in the acrosomal region of spermatozoa with intact acrosome.

**Discussion:**

Taken together, the data present here show that CaMKII and *p*CaMKII localization is dynamic during bovine sperm capacitation and acrosome reaction and that this pattern of localization is affected by heat shock, suggesting that failure in CaMKII signaling is probably involved in the early acrosome reaction observed in heated-shock spermatozoa.

## 1 Introduction

The increase in the body temperature associated with high air humidity changes the metabolic and physiological balance in a process defined as Heat Stress. Heat Stress can modify the microenvironment of the reproductive tract in mammals, affecting their fertility. It can be observed in production animals such as cattle, swine, and sheep that have low fertility in the summer compared to other seasons.

The body temperature in cattle ranges from 36.7°C to 39.1°C, while the testicular temperature in mammals is usually 2°C–6°C lower than body temperature (Kastelic et al., 1999) to allow the testis to support the spermatogenesis properly, in which testis at body temperature impairs the spermatogonia, spermatocytes, spermatids and spermatozoa and function (Nakamura et al., 1987). However, factors such as environmental temperature and relative air humidity can affect spermatogenesis. It can be evidenced by the effect of seasonality on sperm quality in bulls, in which there is a significant reduction in semen volume and sperm concentration and an increase in sperm abnormalities during the summer (Gholami et al., 2011).

Heat stress increases the testicular temperature in cattle, compromising the semen biochemical profile and quality, consequently impairing the sperm fertilizing capacity and embryonic development (Brito et al., 2003; Rao et al., 2015; Cai et al., 2021). Another example of how elevated temperature can impair male fertility is observed in the pathological condition known as varicocele, in which the abnormally dilated veins in the scrotum increase the warm blood flow to the testes affecting the spermatogenesis (Kang et al., 2022). Moreover, testicular heat stress induces germ cell loss, sperm morphological abnormalities and DNA fragmentation, and reduced sperm motility, concentration, and plasma membrane integrity (Yin et al., 1997; Maya-Soriano et al., 2013; Hamilton et al., 2016, 2018; 2019; Houston et al., 2018).

Although several studies have shown that high environmental temperature (heat stress), negatively affects spermatogenesis and consequently semen quality, little is known about the direct effect of heat stress on sperm quality (heat shock). Cows under heat stress conditions have an increase in endogenous temperature and consequently in the entire reproductive tract. Thus, when ejaculated during copulation, the spermatozoa are exposed to high temperatures in the female reproductive tract, which can compromise its quality and the processes of sperm capacitation and acrosome reaction during its journey to reach the oocyte.

Heat shock in bovine sperm has been shown to reduce sperm motility, plasma membrane integrity, and mitochondrial membrane potential, affecting fertilization, cleavage, and preimplantation embryonic development rates (Hoelker et al., 2007; Maya-Soriano et al., 2013; Rahman et al., 2014b, 2014a). Heat shock also induces early acrosome reaction, directly affecting the fertilization rates in cattle (Silva et al., 2017). Although the mechanisms involved in the early acrosome reaction in sperm subjected to heat shock are not known, it has been shown that heat shock affects the organization of the cytoskeleton components in bovine sperm (Silva et al., 2015). Interestingly, both tubulin and actin are involved in the acrosome reaction process (Oikonomopoulou et al., 2009), suggesting that the early acrosome reaction may occur by some mechanism involving tubulin and/or actin.

An intrinsic signaling pathway involving several signaling molecules and the posttranslational modification of these proteins such as phosphorylation by protein kinases plays a key regulatory process in the cellular cytoskeleton network and its associated proteins (Feitosa et al., 2018, 2020; MacTaggart and Kashina, 2021). A protein kinase that has been involved in the acrosome reaction through its regulatory action on cytoskeletal components, in which failure in its activity results in early acrosome reaction is the Ca^2+^/calmodulin-dependent protein kinase (CaMKII), a serine/threonine-specific protein kinase (Shabtay and Breitbart, 2016).

In spermatozoa, CaMKII is related to reactions that mediate dynein activity, consequently regulating sperm motility and flagellar hyperactivation (Smith, 2002). CaMKII is also present in the acrosome, where its activity is responsible for keeping the acrosome membranes in a pre-assembled and paused state for fusion, preventing early acrosome reaction. The pharmacological inhibition of CaMKII significantly increases the spontaneous acrosome reaction, evidencing the protective and/or inhibitory role of CaMKII on the acrosome reaction (Ackermann et al., 2009). However, it is not yet known whether the CaMKII signaling pathway is involved in the early acrosome reaction in bovine spermatozoa subjected to heat stress. Thus, the present work aims to evaluate the dynamics of CaMKII during capacitation and acrosomal reaction in bovine spermatozoa and to assess whether the localization of CaMKII is affected by heat shock.

## 2 Materials and methods

### 2.1 Experimental design

#### 2.1.1 Experiment 1 - dynamics of total and phosphorylated CaMKII during sperm capacitation

To evaluate the dynamics of total and phosphorylated CaMKII during bovine sperm capacitation, spermatozoa were immediately processed after thawed (0 h control) or resuspended at the concentration of 1 × 10^6^ spermatozoa/mL and incubated at 38.5°C for 4 h in a non-capacitation medium (incubation control) or in *capacitation-inducing medium* (treatment). At the end of the incubation, the spermatozoa were submitted to immunofluorescence and evaluated by fluorescence microscopy. Three replicates were performed, and 100 spermatozoa were evaluated per experimental group/replicate.

#### 2.1.2 Experiment 2 - dynamics of total and phosphorylated CaMKII during the acrosome reaction

To evaluate the CaMKII and *p*CaMKII dynamics during the acrosome reaction, spermatozoa were resuspended in the *capacitation*-inducing *medium* at the concentration of 1 × 10^6^ spermatozoa/mL followed by incubation at 38.5°C for 4 h. After that, the spermatozoa were immediately evaluated (capacitated control) or incubated for 1 h in the *capacitation-inducing medium* in the absence (unreacted control) or presence of ionophore calcium (5 mM) and heparin (20 mg/mL) for acrosome reaction induction (treatment). Three replicates were performed, and 100 spermatozoa were evaluated per experimental/replicate.

#### 2.1.3 Experiment 3 – effect of heat shock during sperm capacitation on acrosome reaction and *p*CaMKII localization

To evaluate the effect of heat shock during sperm capacitation on *p*CaMKII localization and acrosome reaction, the spermatozoa were processed immediately after thawing (0 h Control) or resuspended in the capacitation-inducing medium at the concentration of 1 × 10^6^ spermatozoa/mL and incubated for 4 h at 38.5°C (control) or 41°C (heat shock). Four replicates were performed and 100 spermatozoa per experimental group/replicate were evaluated. For each experimental group, the kinetics of the acrosome reaction and *p*CaMKII localization were evaluated at 0 h; 1 h; 2 h; 3 h, and 4 h of incubation. To evaluate the effect of heat shock on total CaMKII localization were analyzed 25 cells per experimental group.

### 2.2 Ethical standards

The experimental procedures performed in the present study were in accordance with the Guidelines for Ethical Principles in Animal Research set forth by the National Council for the Control of Animal Experimentation ‐ CONCEA, and the protocol received Institutional approval (5380040917) from the Ethics Committee in Animal Use (CEUA) of the Federal University of Sao Paulo.

### 2.3 Spermatozoa preparation

Frozen-thawed spermatozoa were centrifuged in Percoll® gradient (90% and 45%) for 5 min at 9.000 × *g*. The pellet was resuspended and washed by centrifugation for 2.5 min at 9.000 × *g* on SP-TALP (Parrish et al., 1988) without NaHCO_3_^−^ and CaCl_2_. After that, the sperm concentration was calculated and diluted at the concentration of 1 × 10^6^ spermatozoa/mL following the experimental groups. For each replicate, two straws of semen from different bulls were randomly selected from a pool of five Holstein bulls.

### 2.4 Incubation and sperm capacitation

To induce *in vitro* capacitation in bovine sperm, the spermatozoa diluted at the concentration of 1 × 10^6^ spermatozoa/mL were incubated for 4 h at 38.5°C under a humidified atmosphere of 5% CO_2_ in non-capacitation (SP-TALP, without NaHCO_3_ and CaCl_2_) or capacitation medium (SP-TALP medium, supplemented with BSA 3 mg/mL and heparin 20 mg/mL).

### 2.5 Acrosome reaction induction

In order to induce the acrosome reaction, bovine spermatozoa were diluted in the *capacitation-inducing medium* at the concentration of 1 × 10^6^ spermatozoa/mL and incubated for 4 h at 38.5°C under a humidified atmosphere of 5% CO_2_. After that, spermatozoa were incubated for 1 h in the absence (control) or presence of ionophore calcium (5 mM) and heparin (20 mg/mL) to induce the acrosome reaction.

### 2.6 Heat shock induction

In order to induce heat shock, bovine spermatozoa were resuspended in 1 mL of the capacitation-inducing medium at 1 × 10^6^ sperm/mL and incubated for 4 h at 41°C under a humidified atmosphere of 7% CO_2_. The control group was incubated in the capacitation-inducing medium at 38.5°C under a humidified atmosphere of 5% CO_2_.

### 2.7 Immunofluorescence

The spermatozoa were adhered to circular coverslips by centrifugation using the CitoSpin at 2800 r.p.m for 10 min. After that, spermatozoa were fixed in 3.7% paraformaldehyde for 30 min at room temperature, permeabilized for 10 min in 0.1% Triton X-100 and blocked in 1% BSA in PBS overnight. After blocking, the spermatozoa were incubated for 1 h at room temperature with the rabbit monoclonal (EP1829y) antibody anti-CaMKII (abcam, ab52476) or rabbit polyclonal antibody anti-phosphorylated (T286) CaMKII (pCaMKII; abcam, ab47565) used at a dilution of 1:100 each. The spermatozoa were then washed with PBS–PVP, and incubated with secondary antibody Alexa Fluor-555 anti-rabbit IgG (Life technologies. A-21429) at a dilution of 1:200 for 1 h at room temperature (RT). Next sperm DNA was counterstained with Hoechst 33342 (5 μg/mL), and the acrosome membrane was labeled with Pisum sativum agglutinin (FITC-PSA; 100 μg/mL). Samples were mounted with vectashield® on glass slides and evaluated under the epifluorescence microscope. The acquired images were analyzed by ImageJ software.

### 2.8 Statistical analysis

The statistical analysis was performed using SigmaPlot 14.0 software (Systat Software, Inc. California, United States). Analysis of variance (ANOVA) assumptions: Normally distributed data and homogeneity of variance were initially evaluated by the Shapiro-Wilk and Brown-Forsythe test respectively. The data that did not meet the ANOVA assumptions (*p*CaMKII dynamics during the acrosome reaction) were square-root transformed. The data from CaMKII and pCaMKII dynamics during the acrosome reaction; from the effect of incubation time and temperature on acrosome reaction and *p*CaMKII localization were analyzed by the Two-Way ANOVA with a *post hoc* Tukey test. The data from the dynamics of CaMKII and *p*CaMKII during sperm capacitation and the data from the effect of heat shock on pCaMKII localization in spermatozoa with intact acrosome were analyzed by One-Way ANOVA with a *post hoc* Tukey test. The chi-square test was used to compare the data from the temperature effect on the localization of total CaMKII. Data are shown as mean (%) ± SD.

## 3 Results

### 3.1 Dynamics of CaMKII during bovine sperm capacitation

**FIGURE 1:**
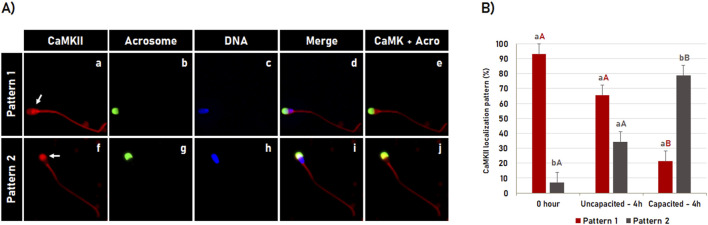
CaMKII dynamic during bovine sperm capacitation. **(A)** Spermatozoa were evaluated after thawing (control 0 h) or after 4 h of incubation in the capacitating or non-capacitating medium. The CaMKII **(a, f)** and acrosome **(b, g)** were evaluated using fluorescence microscopy following fluorescent labeling with rabbit anti-CaMKII antibody and Pisum sativum agglutinin (FITC-PSA), respectively. DNA was labeled with Hoechst 33342 **(c, h)**. Merged images (CaMKII + Acrosome + DNA) are shown in **(d, i)**, while merged images of CaMKII with Acrosome are shown in **(e, j)**. During capacitation, two distinct patterns (P) of CaMKII localization were observed: P1 - High CaMKII concentration at the post-acrosomal region (**a–e**; arrow in a) and P2 - high CaMKII concentration at the acrosomal region (**f–j**, arrow in f). **(B)** Effect of bovine sperm capacitation on CaMKII localization. Results are expressed as the mean (%) ± SME of 3 independent replicates. Lowercase letters represent comparisons between patterns (P1 versus P2) within each treatment (0 h; uncapacitated – 4 h or capacitated – 4 h). Capital letters represent comparisons for each pattern (P1 or P2) among treatments (0 h, uncapacitated – 4 h and capacitated – 4 h). Different letters indicate a significant difference (*P* < 0.05).

The CaMKII dynamics during bovine sperm capacitation were visualized by indirect immunofluorescence based detection, in which the spermatozoa were evaluated immediately after thawing (0 h control) or after 4 h of incubation in the non-capacitating medium (incubation control) or in the capacitation medium (Figure 1A). The immunofluorescence results showed that after thawing (0 h control), CaMKII was observed in the sperm head and tail. Concerning the tail, CaMKII was specifically detected in the middle and in the principal piece of the spermatozoa with intact acrosome membrane. Following 4 h of incubation, the CaMKII localization in the tail was not affected by incubation time or by capacitation. When CaMKII at the sperm head was evaluated, two distinct patterns (P) of CaMKII localization were observed in most spermatozoa (97%): P1 - High CaMKII concentration in the post-acrosomal region (Figures 1a–e; arrow in a) and; P2 - High CaMKII concentration in the acrosomal region (Figures 1f–j, arrow in f). The CaMKII dynamic during sperm capacitation is shown in Figure 1B. In post-thaw sperm (0 h control), most spermatozoa significantly (p < 0.05) showed the pattern 1 (93.1% ± 3.9%). Following 4 h of incubation in the non-capacitation medium, although an increase in the percentage of spermatozoa displaying the pattern 2 was observed, most cells (p < 0.05) were still displaying the pattern 1 (65.7% ± 3.9%). However, this pattern was inverted after 4 h of incubation in the capacitation medium. Most spermatozoa significantly (p < 0.05) displayed the pattern 2 of CaMKII localization (78.7% ± 3.9%), in which sperm capacitation significantly increased (p < 0.05) the percentage of spermatozoa with pattern 2 of CaMKII localization compared to post-thaw sperm (0 h control) and incubated in a non-capacitation medium (incubation control).

### 3.2 Localization of phosphorylated CaMKII during bovine spermatozoa capacitation

**FIGURE 2:**
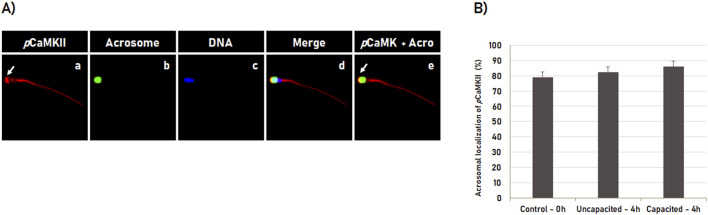
Subcellular localization of phosphorylated CaMKII (*p*CaMKII) during sperm capacitation. **(A)** Bovine sperm were evaluated after thawing (0 h) or after 4 h of incubation in the capacitating or non-capacitating medium. *p*CaMKII **(a)** and acrosome **(b)** were evaluated by fluorescence microscopy using rabbit anti-phosphorylated-CaMKII (T286) antibody and Pisum sativum agglutinin (FITC-PSA), respectively. The DNA was counterstained with Hoechst 33342 **(c)**. The merged images (*p*CaMKII + Acrosome + DNA) are shown in **(d)**, while the merged images of *p*CaMKII with Acrosome are shown in **(e)**. During capacitation, *p*CaMKII is predominantly observed at the apical acrosome region in sperm with intact acrosome (arrow in **a, e**). **(B)** Sperm capacitation effect on *p*CaMKII localization in bovine sperm. Results are expressed as the mean (%) ± SD of 3 independent replicates.

**FIGURE 3:**
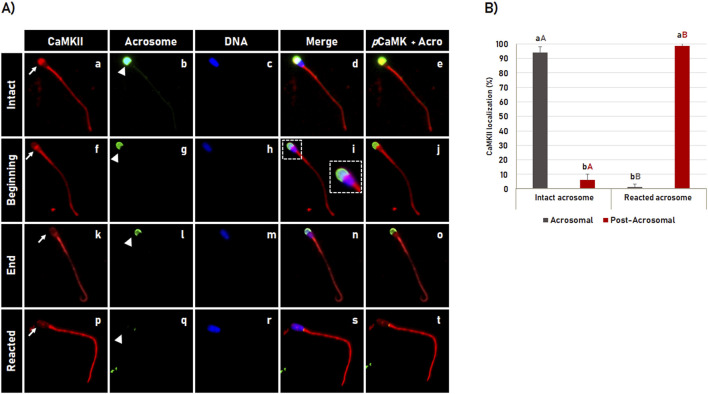
CaMKII localization during acrosomal reaction in bovine sperm. **(A)** After 4 h of incubation in capacitating medium, the acrosome reaction was induced by Ca^2+^ and heparin for 1 h. CaMKII **(a, f, k, p)** and acrosome **(b, g, l, q)** were evaluated by fluorescence microscopy using rabbit anti-CaMKII antibody and Pisum sativum agglutinin (FITC-PSA), respectively. The sperm nucleus was stained with Hoechst 33342 **(c, h, m, r)**. The merged images of CaMKII + Acrosome + DNA are shown in **(d, i, n, s)**, and merged images of CaMKII with acrosome are shown in **(e, j, o, t)**. The CaMKII observed at the acrosomal region of the spermatozoa with intact acrosome **(a–e)**, changes its localization to the post-acrosomal region at the beginning **(f–j)** and the end **(k–o)** of the acrosome reaction until it is no longer observed in spermatozoa with reacted acrosome **(p–t)**. See arrows in **(a, f, k, p)** for CaMKII and arrowheads in **(b, g, l, q)** for acrosome dynamics during the acrosome reaction. Large white square shown in “**i**” is enlargement of the corresponding small box. **(B)** Effect of the acrosome reaction on the CaMKII localization in bovine spermatozoa. Results are expressed as the mean (%) ± SD of 3 replicates. Lowercase letters represent comparisons of CamKII localization (acrosomal versus post-acrosomal) within each acrosomal status (intact or reacted). Capital letters represent comparisons of the same CamKII localization pattern (acrosomal or post-acrosomal) between different acrosomal status (intact versus reacted). Different letters represent a significant difference (p < 0.05).

The localization dynamic of the phosphorylated CaMKII (*p*CaMKII) was performed by immunofluorescence using a specific antibody anti-CaMKII phosphorylated at threonine 286 (T286). For that, spermatozoa were analyzed immediately after thawing (0 h control) or after 4 h incubation in the non-capacitating medium (incubation control) or in the capacitation medium (treatment). Immunofluorescence analyses showed that *p*CaMKII was localized in the middle piece and the intermediate piece of all spermatozoa evaluated (Figures 2A, a–e). The *p*CaMKII was also observed in the head of the spermatozoa with intact acrosome membrane, in which it was localized occupying predominantly the apical region (the leading edge) of the acrosome (Figure 3A, arrow in a and e). However, differently from that observed for CaMKII, no effect (p ˃ 0.05) of the *in vitro* capacitation was observed in the pCaMKII localization pattern, in which the pCaMKII localization was not affected (p ˃ 0.05) by spermatozoa incubation in non-capacitation medium (incubation control) as well as capacitation medium when compared to post-thawing (0 h control) spermatozoa (Figure 2B).

### 3.3 Dynamics of CaMKII during the acrosome reaction in bovine spermatozoa

To evaluate the CaMKII dynamic during acrosome reaction, bovine spermatozoa were *in vitro* capacitated for 4 h followed by acrosome reaction induction for 1 h. After a total of 5 h incubation, the CaMKII localization and acrosome membrane integrity were evaluated by immunofluorescence. It was observed that the pattern of CaMKII localization in the acrosomal region followed the same pattern observed for the acrosome membrane integrity (Figure 3A). After acrosome reaction induction, CaMKII was observed localized in the acrosomal region in most spermatozoa with intact acrosome membrane (94.4% ± 2.4%). As the spermatozoa began to undergo the acrosome reaction, as indicated by the fluorescence intensity of the acrosome labeled with FITC-PSA (green color in Figure 3A, arrow in e, j, o, t), the CaMKII gradually translocates from acrosomal to the post-acrosomal region (Figures 3A, a, f, k), until it is no longer observed in the sperm head in most spermatozoa (98.7% ± 1.1%) with reacted acrosome (Figures 3A,p). The acrosome reaction significantly (p < 0.05) changed the acrosomal localization of CaMKII in spermatozoa with intact acrosome to post-acrosomal localization in spermatozoa with reacted acrosome as shown in Figure 3B.

### 3.4 Localization of phosphorylated CaMKII during the acrosome reaction

**FIGURE 4:**
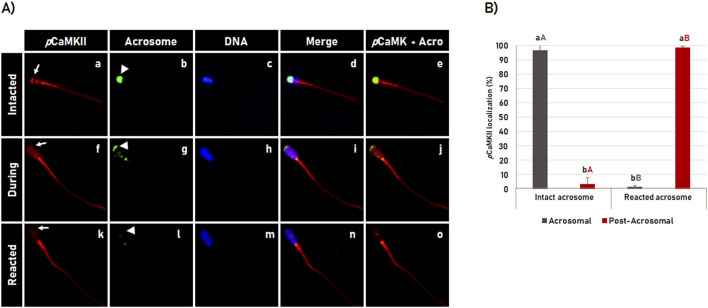
Dynamics of the phosphorylated CaMKII (*p*CaMKII) localization during acrosome reaction in bovine sperm. **(A)** After 4 h of incubation in capacitating medium, the acrosome reaction was induced with Ca^2+^ and heparin for 1 h. *p*CaMKII **(a, f, k)** and acrosome **(b, g, l)** were evaluated by fluorescence microscopy using anti-phosphorylated-CaMKII (T286) antibody and Pisum sativum agglutinin (FITC-PSA), respectively. DNA was stained with Hoechst 33342 **(c, h, m)**. The merged images of CaMKII + Acrosome + DNA are shown in **(d, i, n)** while merged images of CaMKII + Acrosome are shown in **(e, j, o)**. The *p*CaMKII observed at the apical region in intact acrosome **(a–e)**, decreases its apical localization during the acrosome reaction **(f–j)**, until it is no longer observed at the acrosomal region in reacted acrosome spermatozoa **(k–o)**. See arrows in **(a, f, k)** for *p*CaMKII and arrowheads in **(b, g, l)** for acrosome dynamics during the acrosome reaction. **(B)** Effect of the acrosomal reaction on the *p*CaMKII localization in bovine sperm. Results are expressed as the mean (%) ± SD of 3 replicates. Lowercase letters indicate comparisons of *p*CamKII localization within each acrosomal state (intact or reacted). Capital letters indicate comparisons of the same *p*CamKII localization pattern (acrosomal or post-acrosomal) between different acrosomal status (intact versus reacted). Different letters indicate a significant statistical difference (p < 0.05).

The localization pattern of CaMKII phosphorylated at T286 (*p*CaMKII) during acrosome reaction was similar to the pattern observed for total CaMKII (Figure 3), in which *p*CaMKII was observed localized in the apical area of the sperm head in most spermatozoa with intact acrosome membrane (96.7% ± 2.7%). During the acrosome reaction process, *p*CaMKII translocates from the apical region of the acrosome (Figures 4A, a–e) throughout the sperm head (Figures 4A, f–j) until it is no longer observed in the apical region of the sperm head (Figures 4A, k–o) in the most acrosome-reacted spermatozoa (98.6% ± 0.6%), where the apical pattern of *p*CaMKI observed in spermatozoa with intact acrosome, significantly (p < 0.05) changed to the post-acrosomal pattern in acrosome-reacted spermatozoa (Figure 4B).

### 3.5 Effect of heat shock during sperm capacitation on acrosome membrane integrity

**FIGURE 5:**
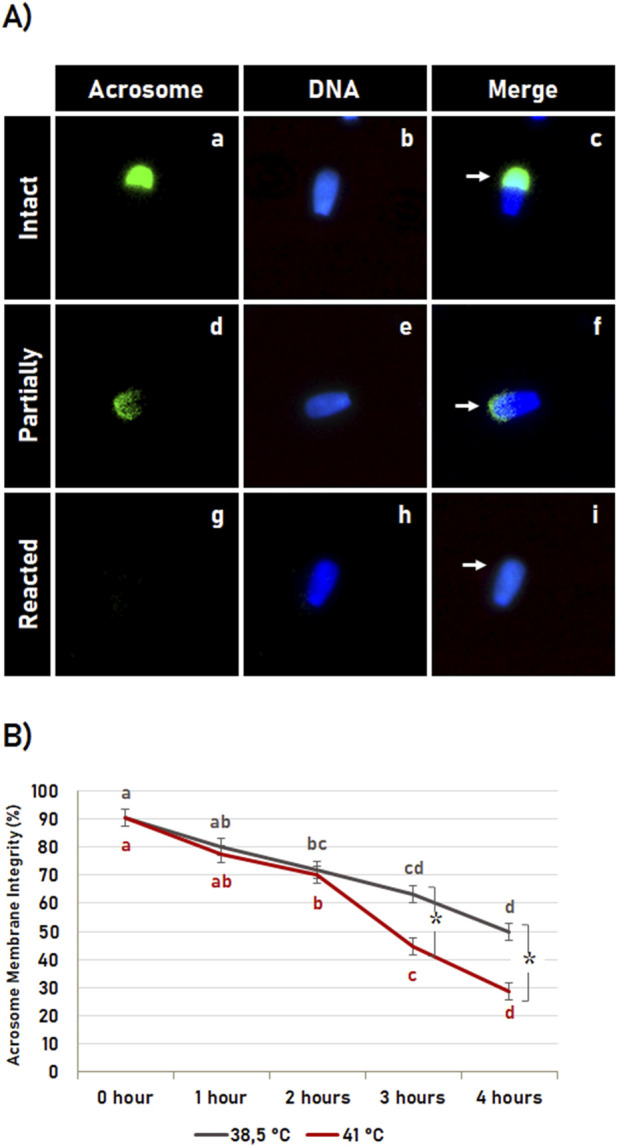
Heat shock effect on acrosome membrane integrity. **(A)** Representative image of bovine sperm with intact acrosome (**a–c**, arrow in **c**), partially reacted (**d–f**, arrow in **f**), and reacted (**g–I**, arrow in **i**). **(B)** Effect of incubation time and heat shock on acrosome membrane integrity during sperm capacitation. Data expressed as the mean (%) ± SD of 4 replicates. Letters indicate comparisons among the different time-point within each temperature (38.5°C or 41°C). Symbol (*) indicates a comparison of each time-point (0 h, 1 h, 2 h, 3 h or 4 h) between the temperatures (38.5°C versus 41°C). Different letters and symbol (*) indicate a significant difference (p < 0.05).

The acrosome membrane integrity (Figure 5A) was significantly affected (p < 0.05) by the incubation time, in which *in vitro* sperm capacitation at 38.5°C as well as at 41°C resulted in an increased acrosome reaction in a time-dependent way (Figure 5B). The acrosome membrane integrity was also affected by the temperature (p < 0.05), where heat shock resulted in an early acrosome reaction. However, the heat shock effect on early acrosome reaction was observed only after 2 h of incubation, where *in vitro* capacitation at 41°C for 3 and 4 h resulted in a higher acrosome reaction rate compared to *in vitro* capacitation at 38.5°C (Figure 5B).

### 3.6 Heat shock effect on CaMKII localization during sperm capacitation

**FIGURE 6:**
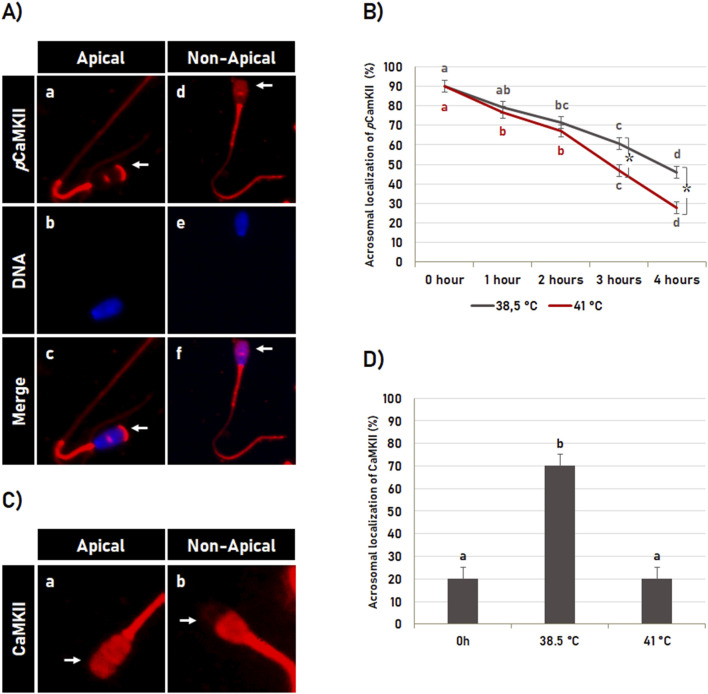
Effect of heat shock on CaMKII localization. **(A)** Illustrative image of bovine sperm with *p*CaMKII localized (**a–c**; see arrow in **a** and **c**) or not (**d–f**; see arrow in **d** and **f**) at the apical region of the acrosome. **(B)** Effect of the incubation time and heat shock on *p*CaMKII localization during sperm capacitation. Data are expressed as the mean (%) ± SD of 4 replicates. Different letters and symbol (*) indicate a significant difference (p < 0.05). Letters indicate comparisons among the different time-point within each temperature (38.5°C or 41°C). Symbol (*) indicates a comparison of each time-point (0 h, 1 h, 2 h, 3 h or 4 h) between the temperatures (38.5°C versus 41°C). **(C)** Heat shock effect on CaMKII localization in bovine sperm. Representative image of bovine sperm with CaMKII localized (arrow in a) or not (arrow in b) at the acrosomal region. **(D)** Effect of heat shock during capacitation on CaMKII localization at the acrosomal region of bovine sperm. Data are expressed as the mean (%) ± SD of 25 cells. Different letters indicate a significant difference (p < 0.05).

As above-mentioned, the incubation time significantly affected (p < 0.05) the *p*CaMKII localization (Figure 6A). In post-thaw semen (0 h control), the CaMKII phosphorylated at T286 was observed localized in the apical region of the sperm head in most spermatozoa. However, the percentage of spermatozoa displaying this pattern of *p*CaMKII localization decreased gradually during *in vitro* capacitation at 38.5°C as well as at 41°C (Figure 6B). However, the pattern of *p*CaMKII localization was also significantly (p < 0.05) affected by the temperature. Similarly to that observed in the integrity of the acrosome membrane, heat shock also affected *p*CaMKII localization only after 2 h of incubation, in which *in vitro* sperm capacitation for 3 and 4 h at 41°C reduced the percentage of spermatozoa with *p*CaMKII localized at the apical region of the head compared to spermatozoa incubated at 38.5°C (Figure 6B).

Heat shock also affected the localization of total CaMKII (Figure 6C). *In vitro* capacitation for 4 h at 38.5°C increased the percentage of spermatozoa with CaMKII localized at the acrosomal region (Figures 6C, a) and consequently decreased (p < 0.05) the percentage of the spermatozoa with CaMKII localized at the post-acrosomal region (Figures 6C, b) compared to post-thaw spermatozoa (0 h control; Figure 6D). However, the *in vitro* capacitation effect on CaMKII localization at the acrosomal region was significantly inhibited (p < 0.05) by heat shock, in which the percentage of spermatozoa with CaMKII at the acrosomal region was similar to the percentage observed in post-thawed spermatozoa (0 h control; Figure 6D).

**FIGURE 7:**
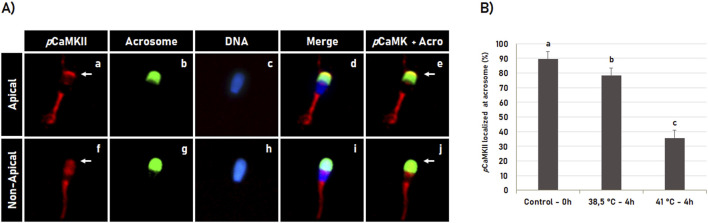
Heat shock effect on phosphorylated CaMKII (*p*CaMKII) localization in bovine sperm with intact acrosome during sperm capacitation. **(A)** Bovine sperm were evaluated after thawing (0 h) or after 4 h of incubation in the capacitating medium at 38.5°C and 41°C. *p*CaMKII **(a, f)** and acrosome **(b, g)** were evaluated by fluorescence microscopy using anti-phosphorylated-CaMKII (T286) antibody and Pisum sativum agglutinin (FITC-PSA), respectively. Hoechst 33342 was used to counterstain DNA **(c, h)**. The merged images of *p*CaMKII + acrosome + DNA is shown in **(d, i)**, while merged images of *p*CaMKII + Acrosome are shown in **(e, j)**. During capacitation, sperm with intact acrosome have *p*CaMKII localized (**a–e**, arrow in **a**, **e**) or not (**f–j**, arrow in **f**, **j**) at the acrosome apical region. **(B)** Effect of heat shock on CaMKII acrossomal localization in bovine sperm with intact acrosome or acrosome reacted following incubation at 38.5°C and 41°C for 4 h. Data are expressed as the mean (%) ± SD of 4 replicates. Different letters indicate a significant difference (p < 0.05).

During sperm capacitation, most spermatozoa with intact acrosome have *p*CaMKII localized in the apical region of the head (Figures 7A, a–e). However, this pattern was significantly (p < 0.05) affected by heat shock during *in vitro* capacitation (Figures 7A, f–j). Sperm capacitation at 41°C significantly (p < 0.05) reduced the percentage of spermatozoa with pCaMKII localized at the apical region of the spermatozoa with intact acrosome membrane compared to the post-thawed spermatozoa and spermatozoa capacitated at 38.5°C (Figure 7B). Data are suggestive that *p*CaMKII translocation from the apical region of the sperm head during heat shock precedes the early acrosome reaction during sperm capacitation.

## 4 Discussion

The Ca^2+^/calmodulin-dependent protein kinase II (CaMKII) is part of the signaling pathway involved in the regulation of sperm motility and flagellar hyperactivation as well as in the inhibition of the acrosomal membrane fusion, thus avoiding early acrosome reaction (Smith, 2002; Ackermann et al., 2009). Herein was observed that the dynamics of the CaMKII localization during sperm capacitation and acrosome reaction are consistent with its physiological function in these processes. In addition, the CaMKII localization was affected by heat shock. It was observed an association between early acrosome reaction and change in the pattern of CaMKII localization during sperm capacitation, suggesting that the early acrosome reaction observed in heat shocked spermatozoa is mediated by CaMKII signaling.

Assessing the dynamics of CaMKII subcellular localization during sperm capacitation, CaMKII was observed localized in the post-acrosomal region in most of the post-thawed spermatozoa (0 h control) and spermatozoa incubated for 4 h in the non-capacitation medium (incubation control). However, sperm capacitation induced the CaMKII translocation from post-acrosomal to the acrosomal region in bovine spermatozoa with intact acrosome. The dynamics of CaMKII localization observed here aligns with its function in inhibiting the acrosome reaction (Ackermann et al., 2009), in which the CaMKII translocation to the acrosomal region during capacitation would be involved in its function to inhibit early acrosome reaction, ensuring that it occurs at the appropriate time.

While sperm capacitation affected the total CaMKII localization, no effect on phosphorylated CaMKII (*p*CaMKII) localization was observed, in which *p*CaMKII was observed in the apical region of the acrosome in capacitated as well as in non-capacitated spermatozoa. CaMKII has catalytic and regulatory domains, where the binding of Ca^2+^/calmodulin to the regulatory domain decreases the auto-inhibition, activating the CaMKII (Makhinson et al., 1999). CaMKII also autophosphorylates at T286, making the kinase constitutively active (Makhinson et al., 1999). Autophosphorylation at T286 transforms CaMKII in one of the ligands with a higher affinity for calmodulin within the cell (Meyer et al., 1992) and renders the CaMKII activity partially autonomous and independent of Ca^2+^ (Coultrap et al., 2010). Autonomous CaMKII can be significantly stimulated by Ca^2+^/calmodulin, since the T286 phosphorylation increases the CaMKII affinity for Ca^2+^/calmodulin, increasing their activity (Coultrap et al., 2012).

Here we observed that CaMKII translocates to the acrosomal region during capacitation, where it will perform its inhibitory role in the acrosome reaction (Ackermann et al., 2009). In this way, it is expected that CaMKII translocate from the acrosomal region during the acrosome reaction, releasing the acrosome from its inhibitory role, to allow the acrosome reaction. Confirming that, during the acrosome reaction, the total and phosphorylated CaMKII was no longer observed in the acrosomal region of the spermatozoa. Moreover, we observed that spermatozoa with acrosome partly intact did not have CaMKII and *p*CaMKII in the acrosomal region, suggesting that the translocation of CaMKII from the acrosomal region precedes the reaction and not the opposite.

One of the mechanisms of CaMKII action occurs through the PDZ domain of MUPP1 protein, which prevents early acrosome reaction regulating the acrosomal membrane fusion (Heydecke et al., 2006; Ackermann et al., 2008). In addition, CaMKII activates the Pyk2-PI3K pathway (Avraham et al., 2000; Fan et al., 2005; Heidinger et al., 2002; Montiel et al., 2007; Rocic et al., 2001), involved in the capacitation and acrosome reaction events by its action on actin filament polymerization (Fisher et al., 1998; Etkovitz et al., 2007; Jungnickel et al., 2007; Shabtay and Breitbart, 2016). The actin filaments are organized during capacitation, with constant polymerization and depolarization capacity, essential for capacitation and acrosome reaction in mammals (Brener et al., 2003). Interestingly, inhibition of CaMKII using KN-93 or W-7 resulted in a significant reduction in the actin filament level and in an increase in early acrosome reaction (Shabtay and Breitbart, 2016). In addition, the acrosome reaction is modulated by actin through mechanisms involving PKA and Src (Brener et al., 2003). In turn, inhibition of PKA and Src significantly decreases the CaMKII phosphorylation/activation (Etkovitz et al., 2009). These results demonstrate that CaMKII inhibits the early acrosome reaction by regulating the actin filaments. Besides, CaMKII participates in the maintenance of sperm motility through actin polymerization (Ignotz and Suarez, 2005; Schlingmann et al., 2007; Shabtay and Breitbart, 2016).

In the present study, the incubation time during sperm capacitation affected similarly the integrity of the acrosome membrane and the acrosomal localization of *p*CaMKII. Likewise, we also observed that heat shock exposure during bovine sperm capacitation increased the early acrosome reaction and affected the acrosomal localization of *p*CaMKII. However, the heat shock effect on acrosome integrity as well as on *p*CaMKII localization was observed only after 2 h of incubation. Similarly, recently was demonstrated that heat shock affected sperm motility only after 2 h of incubation (Silva et al., 2015; da Silva et al., 2022), suggesting that a short period of heat shock has no negative effect on bovine spermatozoa quality. Taken together, our data show an association between acrosome reaction and acrosomal localization of *p*CaMKII, suggesting that the increase in early acrosome reaction observed in bovine spermatozoa exposed to heat shock may be mediated, at least in part, by a change in CaMKII signaling. Corroborating with that, we also observed that heat shock decreased the percentage of spermatozoa with *p*CaMKII localized in the apical region of the intact acrosome. As the *p*CaMKII translocation from the acrosomal region precedes the acrosome reaction, this result suggests that heat shock results in early acrosome reaction by affecting the acrosomal localization of *p*CaMKII.

The mechanisms involved in CaMKII-mediated early acrosome reaction in bovine spermatozoa exposed to heat shock are not known yet. However, since CaMKII inhibits acrosome reaction through actin filaments regulation (Fan et al., 2005; Montiel et al., 2007; Shabtay and Breitbart, 2016) and that actin filaments are affected by heat shock in other cell types (Fan et al., 2016; Malerba et al., 2010), the CaMKII involvement in early acrosome reaction observed in heat-shocked spermatozoa may occur via actin filaments. Recently was demonstrated that heat shock affected the organization of cytoskeletal components such as tubulin and dynein in bovine spermatozoa (Silva et al., 2015). Although the effect of heat shock on actin filaments in spermatozoa is not known yet, it was demonstrated that heat shock caused the disruption, disorganization, and absence of actin filaments in bovine oocytes (Roth and Hansen, 2005; Rodrigues et al., 2016).

Actin filaments are observed in the acrosomal space, between the plasma and outer acrosomal membrane. During the acrosome reaction, phospholipase C (PLC) activation promotes the opening of the Ca^2+^ channel in the outer acrosomal membrane, increasing the intracellular Ca^2+^ concentration ([Ca^2+^]_i_) to break down actin filaments between the membranes (Spungin et al., 1995; Breitbart, 2002), allowing its fusion and consequent acrosome reaction. Inhibition of CaMKII causes the activation of PP1, leading to PKC activation which mediates the acrosome reaction through its action in actin filaments (Spungin et al., 1995; Rotfeld et al., 2014). However, PKC inhibition in bovine sperm exposed to heat shock was not able to prevent the early acrosome reaction (Silva et al., 2017), suggesting that if the actin filaments are evolved in early acrosome reaction in heat-shocked spermatozoa, they are regulated by the CaMKII pathway or by PKC-independent pathway.

## 5 Conclusion

In conclusion, the dynamic localization of CaMKII and *p*CaMKII during bovine sperm capacitation and acrosome reaction is in agreement with their physiological inhibitory role on the acrosome, preventing early acrosome reaction. In addition, heat shock reduced in a similar way the acrosome membrane integrity and *p*CaMKII localization in the acrosomal region, demonstrating an association between early acrosomal reaction and changes in CaMKII localization/activity during heat shock. Heat shock induced the *p*CaMKII translocation from the acrosomal region of spermatozoa with intact acrosome, releasing the acrosome from its inhibitory action, allowing the acrosome to undergo the reaction. Taken together, the data presented here suggest that the early acrosomal reaction in bovine spermatozoa under heat shock may be at least in part mediated by changes in the CaMKII signaling pathway.

## Data Availability

The raw data supporting the conclusions of this article will be made available by the authors, without undue reservation.
